# GENICULAR NERVE ABLATION IN KNEE OSTEOARTHRITIS: A RANDOMIZED PROSPECTIVE STUDY

**DOI:** 10.1590/1413-785220253303e289218

**Published:** 2025-08-18

**Authors:** João Vital Arthur Maradona Oliveira Dias, Vitor Ricardo Alves Pereira de Moraes, Matheus Trindade Bruxelas de Freitas, Matheus Manolo Arouca, Guilherme Pereira Ocampos, Olavo Pires de Camargo, Márcia Uchoa de Rezende

**Affiliations:** 1 Universidade de Sao Paulo, Faculdade de Medicina, Hospital das Clinicas, Instituto de Ortopedia e Traumatologia (HC-FMUSP), São Paulo, SP, Brazil.

**Keywords:** Osteoarthritis, Knee, Pain, Phenol, Radiofrequency Ablation, Osteoartrite do Joelho, Dor, Fenol, Ablação por Radiofrequência

## Abstract

**Objective:**

To document the effects of genicular nerve ablation in patients with severe knee osteoarthritis (OAJ) at 1 and 3 months.

**Methods:**

Prospective, randomized, and controlled trial with 35 patients with grade IV knee osteoarthritis according to Kelgren & Lawrence, undergoing genicular nerve ablation with pulsed radiofrequency (PRF) or phenol. Outcomes were assessed at baseline, 1 month, and 3 months using the Numeric Rating Scale (NRS), the Knee Injury and Osteoarthritis Outcome Score (KOOS), the 30-Second Chair Stand Test (TSL30), and the Timed Up and Go (TUG) test.

**Results:**

The groups were similar at baseline. Both groups improved similarly up to 3 months (p<0.001). NRS decreased by an average of 30% from baseline to all subsequent assessments (p<0.05). TUG decreased between baseline and one month post-procedure (p=0.001), while TSL30 increased from baseline to one and three months (p<0.05). The average behavior of patients was equal between groups over the evaluations for all scores (p Interaction > 0.05). None of the scores differed between groups, regardless of the evaluation period (p Group > 0.05).

**Conclusions:**

Genicular nerve ablation in patients with severe OAJ can provide lasting improvements in pain, function, and quality of life. Both methods, phenol and radiofrequency, are effective. ***Level of Evidence I; Controlled Randomized Clinical Trial***.

## RESUMO

**Objetivo:**

Documentar os efeitos da ablação dos nervos geniculares em pacientes com osteoartrite grave do joelho (OAJ) com 1 e 3 meses.

**Métodos:**

Ensaio prospectivo, randomizado e controlado com 35 pacientes com osteoartrite de joelho grau IV de Kelgren & Lawrence, submetidos à ablação dos nervos geniculares com radiofrequência pulsada (RFP) ou fenol. Os desfechos foram avaliados no início, após 1 e 3 meses, utilizando a Escala de Categoria Numérica (ECN), o Knee Injury and Osteoarthritis Outcome Score (KOOS), o Teste Sentar e Levantar em 30 segundos (TSL30) e o "Timed up and go" (TUG).

**Resultados:**

Os grupos eram similares na inclusão. Ambos melhoraram de forma semelhante até 3 meses (p<0,001). A ECN diminuiu em média 30% do início até todas as avaliações subsequentes (p<0,05). O TUG diminuiu entre o início e um mês pós-procedimento (p=0,001), enquanto o TSL30 aumentou do início até um e três meses (p<0,05). O comportamento médio dos pacientes foi igual entre os grupos ao longo das avaliações para todos os escores (p Interação > 0,05). Nenhum dos escores diferiu em média entre os grupos, independentemente do momento de avaliação (p Grupo > 0,05).

**Conclusões:**

A ablação dos nervos geniculares em pacientes com OAJ grave pode proporcionar melhorias duradouras na dor, função e qualidade de vida. Ambos os métodos, fenol e radiofrequência, são igualmente eficazes. ***Nível de Evidência I; Ensaio Clínico Randomizado Controlado***.

## INTRODUCTION

Osteoarthritis is the most common form of arthritis, occurring symptomatically in the knees of about 10% of men and 15% of women over the age of 60.^1^ It is characterized by the progressive degradation of articular cartilage, and its main recognized outcomes are pain, limitation of activities, and mobility.^2^

The management of pain associated with knee osteoarthritis is a clinical challenge, especially in patients who do not respond adequately to conservative treatments such as anti-inflammatory medications, physical therapy, and lifestyle modifications. In this context, minimally invasive procedures have emerged as promising alternatives for pain control, offering symptomatic relief with lower morbidity compared to more invasive surgical interventions. It is essential to keep in mind that the treatment of OA is not stepped but multimodal. The patient should be educated about the disease and encouraged to take an active role in the treatment. The disease has no cure, but it can be controlled through diet, physical exercises, the use of orthoses, and the administration of medications.^3^

Among these procedures, genicular nerve ablation has gained prominence. It is a procedure to interrupt the pain signaling from the knee to the brain by destroying the nerves. Ablation can be performed using different methods, including the application of phenol and radiofrequency. Both methods effectively reduce chronic knee pain and improve the function and quality of life of patients with knee osteoarthritis who are not candidates for or have failed conservative treatment.^4^

Phenol is a well-established neurolytic chemical agent, acting through a demyelinating and possibly ischemic effect.^5–6^ Its use does not require expensive equipment, and genicular ablation can be performed with few resources.^7^ On the other hand, radiofrequency ablation works by transforming energy into heat, destroying neural tissue.^8^

The aim of this randomized study is to analyze the comparative efficacy of genicular nerve ablation using phenol or radiofrequency in patients with severe knee osteoarthritis. By investigating functional outcomes, we hope to provide substrates that can guide clinical practice and improve pain management in patients with this debilitating condition.

## MATERIALS AND METHODS

This study was conducted by the Osteometabolic Diseases Group of the Department of Orthopedics and Traumatology at the Hospital das Clínicas, University of São Paulo. Patients with knee osteoarthritis were selected and randomized into 2 groups to undergo genicular nerve ablation using radiofrequency (group 1) or phenol (group 2). The study was approved by the University Ethics Committee, under protocol number 1574. The Informed Consent Form was signed by all study participants.

Inclusion criteria used: men and women diagnosed with severe unilateral or bilateral knee osteoarthritis, with a formal indication for unilateral or bilateral total knee arthroplasty, who had a pain score greater than 6 on the Numerical Pain Scale (NPS) and a positive response to the genicular nerve anesthetic block test, in addition to the ability to read, understand, and respond to the questionnaires. The exclusion criteria were: (1) patients who had previously undergone knee arthroplasty on the studied knee, (2) use of a pacemaker, (3) patients who had undergone electroacupuncture, (4) patients who had knee injections in the last 6 months, (5) patients with cognitive, psychiatric, or neurological disorders that interfere with attention, memory, logical reasoning, or comprehension functions, thus preventing them from assimilating the given instructions, (6) use of anticoagulants between the genicular nerve block test and the intervention, (7) recent knee pain of less than 3 months, (8) advanced liver disease, and (9) signs of systemic or local infection before the intervention.

Patients were evaluated regarding personal characteristics and knee functionality scores. The ablation procedures were performed between December 2022 and January 2023, and the patients were followed up after this period.

The personal characteristics of the individuals evaluated were: age, sex, weight, height, and BMI.

Patients were asked to rate their pain using the Numerical Pain Scale (NPS) and were subjected to the functional tests Timed Up and Go (TUG) and the 30-Second Chair Stand Test (30CST). The Knee Injury and Osteoarthritis Outcome Score (KOOS) was also applied to assess knee functionality. Finally, information regarding adverse reactions, analgesic use, and satisfaction with the treatment was obtained. Data were collected at three time points: at the beginning of the treatment, after 1 month, and after 3 months.

The quantitative personal characteristics or initial scores evaluated were described according to groups using summary measures (means, standard deviations, medians, and quartiles) and compared between groups using unpaired t-Student tests. The sex of the patients was described according to groups using absolute and relative frequencies, and the association was verified using Fisher's exact test. The questionnaire scores were described according to groups over the evaluated time points using summary measures and compared between groups and time points using generalized estimating equations (GEE) with normal marginal distribution and identity link function or logarithmic link function, assuming an autoregressive correlation matrix of the first order (AR(1)) between the evaluation moments for all analyses. The analyses were followed by Bonferroni multiple comparisons to verify where the differences occurred between the groups and evaluation moments. The analyses were performed using IBM-SPSS for Windows version 22.0 and tabulated using Microsoft-Excel 2013 software, with tests conducted at a significance level of 5%.

## RESULTS

During the period from December 2022 to January 2023, 35 patients were treated in a blinded and randomized manner with the genicular nerve ablation procedure using either radiofrequency (study group – 17 individuals) or phenol (control group – 18 individuals), adhering to the study's inclusion and exclusion criteria.

Table 1 shows that the personal characteristics of the patients were similar between the groups, indicating that the groups are comparable.

**Table 1:** Description of personal characteristics by groups and results of statistical tests.

Variable	Group		Total (N = 35)	p
	Study (N = 17)	Control (N = 18)		
Age (years)				0.430**
Average ± SD	63.9 *±* 8.8	61.3 *±* 10	62.6 *±* 9.4	
median (p25; p75)	66 (58; 69.5)	60 (53; 69.3)	65 (56; 69)	
**Gender, n (%)**				**0.146**
Female	10 (58.8)	15 (83.3)	25 (71.4)	
Male	7 (41.2)	3 (16.7)	10 (28.6)	
**Weight (kg)**				**0.267** **
Average *±* SD	92.3 *±* 17.4	85.7 *±* 17.3	88.9 *±* 17.4	
median (p25; p75)	91.9 (79.3; 107.1)	81.8 (72.8; 93.8)	86 (75.5; 96.2)	
**Hight (cm)**				**0.414** **
Average ± SD	162 *±* 10.9	159.3 *±* 8	160.6 *±* 9.5	
median (p25; p75)	162 (153; 169.5)	159 (154.5; 163.5)	160 (153; 166)	
**BMI (Kg/m²)**				**0.531** **
Average ± SD	35.3 *±* 6.6	33.9 *±* 7	34.6 *±* 6.7	
median (p25; p75)	34.4 (32.2; 39)	32.6 (30.5; 35.9)	33.4 (31.3; 38.2)	

Exact Fisher test;

** Unpairing t-Student test.

The patients were evaluated at a pre-intervention/initial time point regarding the NRS, KOOS score, and physical performance tests (TUG and 30s-STS). The KOOS score was evaluated separately in its 5 pillars (Symptoms + Stiffness, Pain, Daily Activities, Sports and Leisure Activities, and Quality of Life) as well as overall, with no significant differences found between the groups at the initial time point, as recorded in Table 2. The initial NRS recorded was also not different between the groups. Therefore, the groups presented similar symptomatology and activity levels at the beginning of the study, making them comparable.

**Table 2:** Description of scores assessed at baseline and outcomes by groups and results of statistical tests.

Variable	Group		Total (N = 35)	p
	Study (N = 17)	Control (N = 18)		
ECN				0.253**
Average *±* SD	9.4 *±* 0.8	9.1 *±* 1	9.2 *±* 0.9	
median (p25; p75)	10 (9; 10)	9 (8; 10)	10 (8; 10)	
**TUG**				**0.949** **
Average *±* SD	17.5 *±* 9.3	17.3 *±* 8.2	17.4 *±* 8.6	
median (p25; p75)	14 (11.8; 17.4)	14.3 (11.6; 18.7)	14.3 (11.8; 17.2)	
**TLS30S**				**0.343** **
Average *±* SD	7 *±* 2.3	6.3 *±* 1.9	6.6 *±* 2.1	
median (p25; p75)	7 (5; 9)	6 (5; 8)	7 (5; 8)	
**KOOS General**				**0.769** **
Average *±* SD	25.8 *±* 17	24.3 *±* 12.5	25 *±* 14.6	
median (p25; p75)	24 (10.5; 41)	27 (10; 35)	26 (10; 37)	
**KOOS Symptoms+Rigidity**				**0.318£**
Average *±* SD	38.7 *±* 26.7	29.8 *±* 13.1	34.1 *±* 21	
median (p25; p75)	50 (9; 61)	32 (20.3; 40.8)	32 (14; 54)	
**KOOS Pain**				**0.684£**
Average *±* SD	24.9 *±* 16.3	27.4 *±* 16.6	26.2 *±* 16.2	
median (p25; p75)	28 (8; 40.5)	34.5 (7.5; 39.8)	33 (8; 39)	
**KOOS Daily Activity**				**0.935£**
Average *±* SD	29.1 *±* 20.8	29 *±* 17.7	29.1 *±* 19	
median (p25; p75)	28 (11; 47)	33.5 (11.5; 41.8)	32 (12; 44)	
**KOOS Sports and Leisure Activity**				**0.613£**
Average *±* SD	6.8 *±* 9.2	4.2 *±* 5.8	5.4 *±* 7.6	
median (p25; p75)	0 (0; 10)	0 (0; 6.3)	0 (0; 10)	
**KOOS Quality of Life**				**0.351£**
Average *±* SD	13.4 *±* 10.6	10.6 *±* 12.8	11.9 *±* 11.7	
median (p25; p75)	13 (0; 22)	6 (0; 13.5)	13 (0; 19)	
**Adverse reaction. n (%)**				**0.697**
No	4 (36.4)	6 (46.2)	10 (41.7)	
Yes	7 (63.6)	7 (53.8)	14 (58.3)	
**Have used any analgesic?. n (%)**				**>0.999**
No	7 (70)	8 (66.7)	15 (68.2)	
Yes	3 (30)	4 (33.3)	7 (31.8)	
**Increased use of analgesics?. n (%)**				**0.495** #
Decreased	8 (80)	8 (66.7)	16 (72.7)	
Unchanged	2 (20)	3 (25)	5 (22.7)	
Increased	0 (0)	1 (8.3)	1 (4.5)	
**Was satisfied?. n (%)**				**0.411** #
Unsatisfied	0 (0)	1 (8.3)	1 (4.5)	
Indifferent	1 (10)	2 (16.7)	3 (13.6)	
Satisfied	8 (80)	6 (50)	14 (63.6)	
Very satisfied	1 (10)	3 (25)	4 (18.2)	

Exact Fisher test;

# Test of the likelihood ratio;

** Unpairing t-Student test; £ Mann-Whitney test

Finally, the degree of patient satisfaction, the presence of adverse reactions, and the use of analgesics were also recorded.

In our study, complications were mainly observed between the first and third weeks, including: eight cases of pain at the puncture site, eight cases of local edema, seven cases of increased local temperature, eight cases of ecchymosis, four cases of paresthesia, and one case of local bleeding. No serious adverse events were observed.

**Figure 1:**
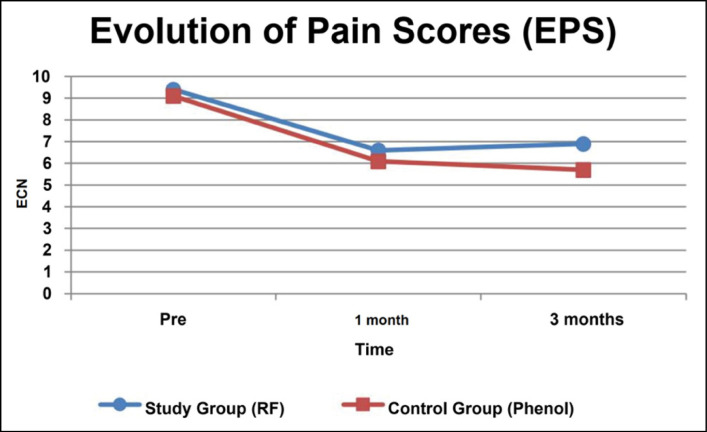
Graph with results of pain assessment using the numeric rating scale (NRS) at the 3 evaluated time points.

Figure 1 presents the results of the NRS evaluation at the 3 time points of the study; the NRS decreased by an average of 30% from the beginning to all subsequent evaluations (p<0.05).

Table 3 shows that the average behavior of the patients was the same between the groups over the evaluation periods for all assessed scores (Interaction p > 0.05), none of the scores differed on average between the groups regardless of the evaluation periods (Group p > 0.05), and almost all scores showed significant average differences over the evaluation periods (Moment p < 0.05), with the exception of KOOS sports and leisure activities and KOOS quality of life (Moment p = 0.052 and Moment p = 0.053, respectively). These findings demonstrate the positive results of the interventions performed, with no significant differences between them (radiofrequency vs. phenol).

**Table 3:** Description of scores by groups and assessment time points and results of comparative tests.

Variable	Study			Control			p Group	p Moment	p Interaction
	Pre	1 month	3 months	Pre	1 month	3 months			
ECN							0.209	<0.001	0.666
Average *±* SD	9.4 *±* 0.8	6.6 *±* 2.2	6.9 *±* 3.1	9.1 *±* 1	6.1 *±* 3.3	5.7 *±* 2.8			
median (p25; p75)	10 (9; 10)	7 (5; 8)	7 (5; 10)	9 (8; 10)	6.5 (3.8; 9)	6 (4.3; 7.3)			
**TUG**							**0.492**	**0.016**	**0.512**
Average *±* SD	17.5 *±* 9.3	14.7 *±* 5.6	15.4 *±* 13.3	17.3 *±* 8.2	13.3 *±* 5.1	12.5 *±* 4.1			
median (p25; p75)	14 (11.8; 17.4)	14 (11.5; 15.9)	12.4 (9.7; 16.7)	14.3 (11.6; 18.7)	10.9 (9.6; 16.9)	10.6 (9.4; 15.5)			
**TLS30S**							**0.286**	**<0.001**	**0.375**
Average *±* SD	7 *±* 2.3	8.6 *±* 2.3	8.9 *±* 2.9	6.3 *±* 1.9	7.5 *±* 2.6	8.7 *±* 1.8			
median (p25; p75)	7 (5; 9)	8 (7; 9.8)	8.5 (6; 11)	6 (5; 8)	7 (6; 9.3)	8 (7; 10)			
**KOOS General**							**0.520**	**<0.001**	**0.206**
Average *±* SD	25.8 *±* 17	39.9 *±* 20.2	41.6 *±* 18.1	24.3 *±* 12.5	41.6 *±* 19.8	48.8 *±* 20.6			
median (p25; p75)	24 (10.5; 41)	35 (27; 59)	43.5 (29.3; 53.5)	27 (10; 35)	42 (30; 52)	45 (33.8; 67.3)			
**KOOS Symptoms+Rigidity** *							**0.834**	**<0.001**	**0.182**
Average *±* SD	38.7 *±* 26.7	54.4 *±* 27.6	54.6 *±* 22.7	29.8 *±* 13.1	54.9 *±* 22.5	61.3 *±* 23.9			
median (p25; p75)	50 (9; 61)	53.5 (33; 75)	54 (44.3; 73)	32 (20.3; 40.8)	61 (39.5; 68)	61 (44.3; 86.8)			
**KOOS Pain** *							**0.463**	**<0.001**	**0.180**
Average *±* SD	24.9 *±* 16.3	43.9 *±* 24.1	46.1 *±* 21.2	27.4 *±* 16.6	44.9 *±* 21.4	55.4 *±* 24.6			
median (p25; p75)	28 (8; 40.5)	44 (26; 62.5)	45.5 (31; 64)	34.5 (7.5; 39.8)	47 (32; 57)	52.5 (37.5; 74.3)			
**KOOS Daily Activity** *							**0.727**	**<0.001**	**0.462**
Average *±* SD	29.1 *±* 20.8	44.6 *±* 20.8	47.4 *±* 21.7	29 *±* 17.7	43.7 *±* 25.9	54.6 *±* 24			
median (p25; p75)	28 (11; 47)	35 (30.5; 61.5)	48 (32.8; 59.3)	33.5 (11.5; 41.8)	38 (29.5; 65.5)	45 (38; 80.5)			
**KOOS Sports and Leisure Activity** *							**0.964**	**0.052**	**0.708**
Average *±* SD	6.8 *±* 9.2	15.6 *±* 20	14.6 *±* 13.2	4.2 *±* 5.8	18.5 *±* 21.7	19.4 *±* 14.7			
median (p25; p75)	0 (0; 10)	5 (0; 25)	12.5 (3.8; 21.3)	0 (0; 6.3)	15 (0; 27.5)	22.5 (3.8; 30)			
**KOOS Quality of Life** *							**0.819**	**0.053**	**0.447**
Average *±* SD	13.4 *±* 10.6	18.5 *±* 16	19.3 *±* 12.8	10.6 *±* 12.8	19.4 *±* 20.5	26.1 *±* 20			
median (p25; p75)	13 (0; 22)	13 (6; 31)	19 (11.3; 25)	6 (0; 13.5)	19 (3; 31)	25 (15.8; 32.8)			

EEG with normal distribution and identity bond function. assuming matrix of correlations AR(1) between the moments;

* Same model with logarithmic bond function.

Table 4 shows that all scores that differed over the evaluation periods represented clinically relevant improvements associated with the treatments performed. This is demonstrated by the significant average improvement from the pre/initial period to the other evaluated moments (p < 0.05). The TUG, in turn, showed a significant average improvement between the initial evaluation and the 1-month evaluation (p = 0.013). These findings corroborate those recorded in Table 3, demonstrating that both interventions were effective in controlling pain and improving quality of life.

**Table 4:** Results of multiple comparisons of scores that differed between assessment time points.

Variable	Comparison		Average difference	Standard error	p	IC (95%)	
	Lower	Superior			
ECN	Pre/Initial -	1 month	2.86	0.48	<0.001	1.72	4.00
	Pre/Initial -	3 months	2.92	0.56	<0.001	1.59	4.26
	1 month -	3 months	0.06	0.50	>0.999	-1.12	1.25
TUG	Pre/Initial -	1 month	3.78	1.33	0.013	0.60	6.97
	Pre/Initial -	3 months	3.56	1.69	0.106	-0.49	7.61
	1 month -	3 months	-0.23	1.38	>0.999	-3.54	3.09
TLS30S	Pre/Initial -	1 month	-1.44	0.36	<0.001	-2.30	-0.59
	Pre/Initial -	3 months	-2.00	0.46	<0.001	-3.10	-0.90
	1 month -	3 months	-0.55	0.37	0.399	-1.43	0.33
KOOS General	Pre/Initial -	1 month	-15.57	2.54	<0.001	-21.66	-9.48
	Pre/Initial -	3 months	-18.89	3.32	<0.001	-26.84	-10.94
	1 month -	3 months	-3.32	2.66	0.638	-9.69	3.06
KOOS Symptoms+Rigidity	Pre/Initial -	1 month	-20.48	3.53	<0.001	-28.93	-12.04
	Pre/Initial -	3 months	-22.81	4.56	<0.001	-33.73	-11.89
	1 month -	3 months	-2.33	3.68	>0.999	-11.13	6.48
KOOS Pain	Pre/Initial -	1 month	-18.35	2.78	<0.001	-24.99	-11.70
	Pre/Initial -	3 months	-22.66	3.75	<0.001	-31.64	-13.67
	1 month -	3 months	-4.31	2.97	0.442	-11.43	2.81
KOOS Daily Activity	Pre/Initial -	1 month	-15.02	3.73	<0.001	-23.96	-6.08
	Pre/Initial -	3 months	-20.64	4.72	<0.001	-31.93	-9.35
	1 month -	3 months	-5.62	3.94	0.462	-15.06	3.82

Multiple comparisons of Bonferroni.

## DISCUSSION

Both methods of genicular nerve ablation proved effective for symptom control and improvement in the quality of life of patients with severe knee osteoarthritis. Comparative analysis showed that there were no significant differences between the groups in terms of knee functionality scores over the evaluation periods (Table 3). The improvement showed a lasting effect in the 3-month follow-up, with no significant differences between the phenol and radiofrequency groups (Table 4). This suggests that both techniques are equally effective in controlling chronic knee pain.

The data also reveal that the majority of patients reported a reduction in the use of analgesics and a high level of satisfaction with genicular ablation (Table 2). Only a small percentage of patients experienced adverse effects, which is consistent with the existing literature that considers the procedure safe.

Knowledge of the anatomy of the genicular nerves is essential for performing ablation procedures. These structures are responsible for the sensory innervation of the knee joint capsule and primarily originate from branches of the femoral nerve. They are found in close relation to the epiphysis of the femur and tibia, and the use of radiological imaging to guide ablation procedures provides a robust foundation for safe and effective clinical practice.^9^

Ablation of the genicular nerves, whether by radiofrequency or phenol, has been used safely, with relatively rare complications (pseudoaneurysms, arteriovenous fistulas, hemarthrosis, patellar osteonecrosis, and chemical neuritis due to the use of phenol), which can be managed with appropriate interventions. The use of fluoroscopy and ultrasound with Doppler function can help minimize risks during the procedure.^7–10^ The use of phenol as an ablation method can be a more accessible alternative in low-resource settings, given its greater cost-effectiveness and lower complexity. The radiofrequency procedure may involve higher costs and require more technology and training.^4^ Regarding the surgical treatment of severe knee osteoarthritis, arthroplasty is considered the gold standard. However, it presents higher risks, costs, and potential complications, along with a slower recovery. In this context, minimally invasive procedures such as genicular nerve ablation can be considered safe and effective, representing an important alternative for patients who are at prohibitive risk for arthroplasty or as a method for pain control and functional improvement in the medium term.^11^

Although the results are promising, it is important to recognize the study's limitations. The relatively small sample size of 35 patients may not be representative of the general population. The 3-month follow-up may be considered short for a chronic progressive disease, and more data should be collected regarding the treatment's effectiveness in the medium and long term. Additionally, the absence of a control group that did not receive interventional treatment may limit the ability to generalize the results.

## CONCLUSION

Genicular nerve ablation in patients with severe knee osteoarthritis can provide lasting improvements in pain, function, and quality of life. Both methods, phenol and radiofrequency, were equally effective.

## References

[1] Iannaccone F, Dixon S, Kaufman A (2017). Radiofrequency ablation of genicular nerves for chronic knee osteoarthritis pain: A retrospective chart review and prospective follow-up study. Pain Physician.

[2] WHO Scientific Group on the Burden of Musculoskeletal Conditions at the Start of the New Millennium (2003). World Health Organ Tech Rep Ser.

[3] Rezende MU, Campos GC, Pailo AF (2013). Conceitos atuais em osteoartrite. Acta Ortopédica Brasileira.

[4] Yildiz G, Perdecioglu GRG, Yuruk D, Can E, Akkaya OT (2023). Comparison of the efficacy of genicular nerve phenol neurolysis and radiofrequency ablation for pain management in patients with knee osteoarthritis. Korean J Pain.

[5] Wood KM (1978). The use of phenol as a neurolytic agent: a review. Pain.

[6] Wu R, Majdalany BS, Lilly M, Prologo JD, Kokabi N (2022). Agents Used for Nerve Blocks and Neurolysis. Semin Intervent Radiol.

[7] Ahmed A, Arora D (2019). Ultrasound-Guided Neurolysis of Six Genicular Nerves for Intractable Pain from Knee Osteoarthritis: A Case Series. Pain Pract.

[8] Choi WJ, Hwang SJ, Song JG, Leem JG, Kang YU, Park PH (2011). Radiofrequency treatment relieves chronic knee osteoarthritis pain: a double-blind randomized controlled trial. Pain.

[9] Franco CD, Buvanendran A, Petersohn JD, Menzies RD, Menzies LP (2015). Innervation of the Anterior Capsule of the Human Knee: Implications for Radiofrequency Ablation. Reg Anesth Pain Med.

[10] Kim SY, Le PU, Kosharskyy B, Kaye AD, Shaparin N, Downie SA (2016). Is Genicular Nerve Radiofrequency Ablation Safe? A Literature Review and Anatomical Study. Pain Physician.

[11] Li G, Zhang Y, Tian L, Pan J (2021). Radiofrequency ablation reduces pain for knee osteoarthritis: A meta-analysis of randomized controlled trials. Int J Surg.

